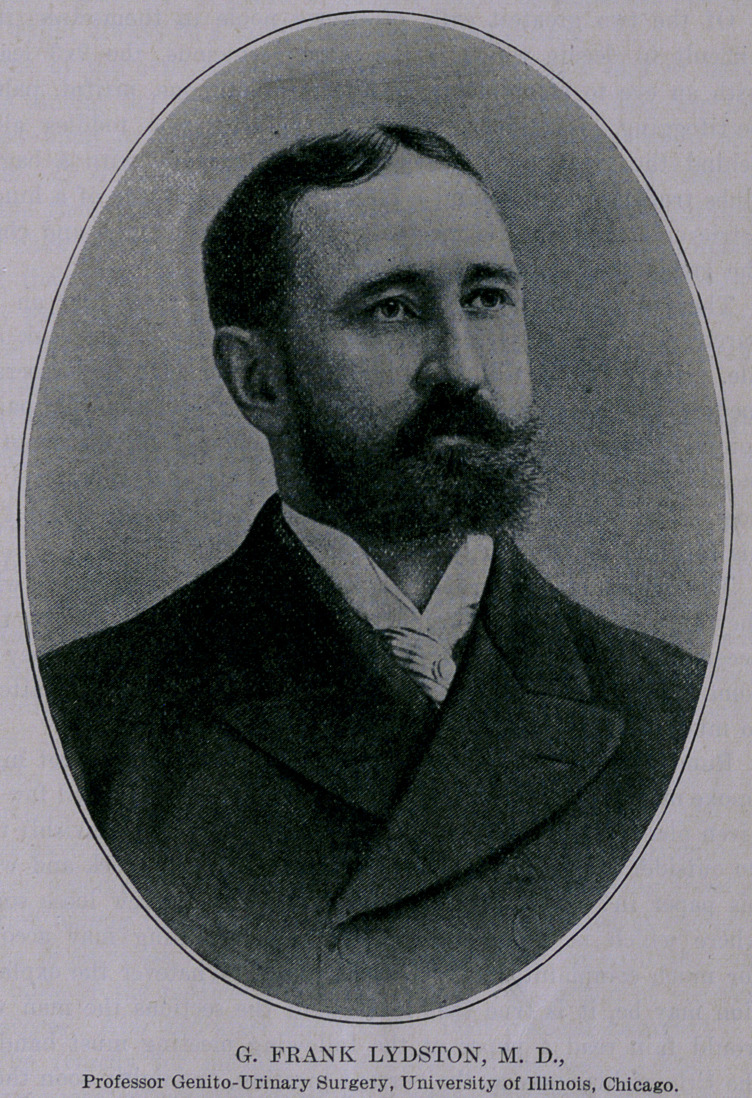# Medical Politics in Epigram and Otherwise

**Published:** 1909-01

**Authors:** G. Frank Lydston

**Affiliations:** Chicago


### TEXAS MEDICAL JOURNAL.

**Established July, 1885.**

F. E. DANIEL, M. D., - - - - Editor, Publisher and Proprietor.

PUBLISHED MONTHLY.—SUBSCRIPTION $1.00 A YEAR.

VOL. XXIV. AUSTIN, JANUARY, 1909. No. 7.

The publisher is not responsible for the views of contributors.

### Original Articles.

For Texas Medical Journal.

### Medical Politics in Epigram and Otherwise.

BY G. FRANK LYDSTON, M. D., CHICAGO.

There are many evils incidental to medical associations, as is true of all organizations. Some of these evils are unavoidable, others not. The larger the association, the greater the evils. Guess which one has the choicest aggregation of evils: "If that red-headed girl in the gallery doesn't behave herself, I'll point her out to the congregation."

Somebody has divided politics into politics, church politics and medical politics. On looking the matter up I find the following suggestive comparisons of the three varieties, viz.: "Rotten, rottener, rottenest." Illustrations are so familiar that to enumerate them would be supererogation.

There is no evil in our medical societies that is not due to sins of omission and commision on the part of the rank and file. Certain fellows will run things for themselves if the sheep do not "watch out." N. B.—They do *not* watch out, and should not be surprised when their "leaders" take the all-hog road to glory and perquisites—material or other. Human nature is ever selfish and moves along the lines of least resistance toward the accomplishment of its ends.

Hero worship is bad business. Out of it groweth tin gods. It is the meat on which our medical Caesars have fed and grown great. Yea, verily, hero worship is the metal out of which medical tin gods are made. Like Frankenstein's monster, the tin god would fain devour his creator. N. B.—And he is doing it, and who shall say him nay?

When the medical sheep fall asleep at the switch funny things happen, e. g., officers of medical societies are elected by acclamation, and great schemes are put in operation for the benefit of the favored few. The silent members wake up the day after, but their weeping and wailing and gnashing of teeth availeth not. Which "it is not to cry, it is to laugh." When the sheep have been inoculated against the medico-political sleeping sickness there be things adoing, and the rank and file will no longer add to the merriment of nations.

The placidity and humbleness of the sheep under the lash is well shown by the fact that the majority of editors of independent medical journals swallow any insulting or oppresive decree that the oligarchy sees fit to cram down their luckless throats. The independent journals will soon be a thing of the past, and when their requiem has been sung the bells will be a-tolling for the independent medical society, for the "gang" is now after that—with the same big stick "organization." Wonder how many "independents" will have nerve enough to quote this article in extenso.

Once upon a time a noble literary lion died, thereby creating a vacuum in circles editorial. A certain, nay, an "uncertain," official board caught a funny biped way out west in the sage brush. None of the works on natural history classified the "find." He looked harmless enough and had no enemies abroad in the East, for lo! he had lived a life of sugar-coated desuetude. His proportions were not unduly conspicuous. His voice was soft and gentle as that of a white buck rabbit singing a ballad to his all-too-willing lady love. And he seemed docile and willing to subsist on "policies." And so they caged him, saying, "Eureka! Here is God's own anointed, against whom can be raised no objecting voice. To h—l with his record. Verily he shall be a mighty editor." And they forthwith stuffed him into the lion's skin.

The bulk of the obscure one grew and grew, till now he looms large and dark on the medical horizon, and his voice became loud and raucous, and he showed his teeth almost fiercely whenever he snarled.

(For the key to part of the above, see Aesop. For the rest, bide a wee.)

*N. B.—There is still a vacuum, for out of nothing nothing cometh.*

A man may not be capable of writing copy for a meal ticket, and correctly addressing two postal cards may completely exhaust his intellectual resources, yet may he obtain "official" recognition and be placed in the chief seat among the mighty—a king among the oreide gods. Even when some recalcitrant Philistine applies

G. FRANK LYDSTON, M. D.,

Professor Genito-Urinary Surgery, University of Illinois, Chicago.

the acid and shows the base metal beneath, the "follow my leaders" will not see, much less act.

The sheep grew frightened, all in their good time, and were sore distraught, having no pin wherewith to prick the mephitic bubble that had arisen in the stagnant pool of medical politics.

And now there be groanings: "How long, O Lord, how long?" And no man can answer, for despite their howls for liberty the sheep most dearly love the collar.

An evil is like unto a bad boy. Correction should be begun early, for by and by he grows too big to thrash, and lo! he punches him who chastiseth.

Of the two greatest evils of causes noble in themselves, it is difficult to decide which is the more pernicious, the 2x3 leader with an eye to emoluments, or the pusillanimous, pitiful, paltry, pettifogging, pestiferous, pinheaded parasite who toddles along behind the great (sic) leader like a little caddy with a bag of clubs trotting along behind a golfer, or plays the rôle of a human retriever and, when his master is not looking, mouths and chews the game, thus showing his mongrel blood.

There never was a one-man power, nor was there ever an oligarchy that was not built up from the stupidity of the rank and file. Some day it will be written: "In the dark undemocratic medical ages agone there was a great medical association that paid a couple of men large salaries to weld the bonds of slavery about its members' necks. The tail finally grew so big that it wagged the dog. Yea, verily, the wrong end ruled. And so the dog bit off his tail and let it merrily wag itself to death."

The officers of certain medical associations are their rulers when they should be rightly considered their servants. By and by they become so puffed up by the flattery of the "humbles" that they become much like a certain fellow's sweetheart, whom he flattered so much that she grew too durned proud to speak to him.

Rumor and "they say" are not very reliable as a rule, yet much smoke means at least a little fire. Is it true that the favored few are given places on the section programs of the A. M. A. so lavishly that no outsider is quick enough to take time by the forelock and work his paper in? One thing is certain, there is a screw loose somewhere which is crying for reform. Overcrowding may account for much complaint, but even this is evil. Whatever the explanation may be, it is true that in some of the sections the man who would fain read a paper at the following meeting must hand in the title of his essay at the current meeting, or mighty soon thereafter. Again, I ask, is it true that favoritism controls? Let the sadly experienced—if there be any such—answer. The "insiders" are not competent witnesses.

Human nature being as it is, the chairmen and secretaries of the various sections might be expected to "take care of their friends." Abuses, partialities and impositions would, therefore, be a natural sequence. How would it do to have a time limit for the submission of papers with a limitation of their number? The allotment of places on the program could satisfactorily be decided by lot—rafflewise. A board of censors might be impartial enough, yet would always be a target for disgruntled critics.

One of the evils of our medical associations is a lack of backbone among the non-office-holding members. When they are imposed upon or bullied they content themselves with feeble protests. If the high and mighty ones vouchsafe them a bone to soothe their troubled spirits they resume the cud of contentment and chew placidly away until their own toes are trodden on again, and they then pipe another feeble wail of protest. Why can't they see that no reforms can be accomplished unless the under dogs stand fast and bark together and back up their barks with a display of teeth—and more if necessary?

A recent incident pertinently illustrates the above. A certain internist volunteered a paper to his section. He was informed that the papers were to be "limited in number" and that those who were to read had "already been selected."

Here was an excellent chance for a fight for principle. Did the victim of the machine take it? Oh, no; as soon as his plaintive squeals were heard at the fountain head he was given a sop, and let into the circle of the sacred chosen of the Lord. He then placidly resumed his cud, caring naught if others were imposed upon, so long as he was "taken care of."

Why did not this gentleman push home his grievance and thus propitiate the star-eyed goddess of medical reform? Surely he must have known that the crumb of comfort thrown so grudgingly to him was merely a sop thrown to the Cerberus of growing dissatisfaction among the rank and file. And yet, he may be as egotistic as he is spineless. If so, he probably accepted the crumbs from the table of the medical Dives as a belated recognition of his own greatness. Alas and alack and a day! Poor medical Lazarus!

The remedy for medical society evils is not easy to find. A galvano-medical-political shock might turn the trick, if the members would only wake up. An injection of medico-socialistic serum into the mighty association's glutei might help some—or is there any relief for society trypanosomiasis?

Some men have panaceas for our Old Man of the Sea, and fondly believe they can unhorse him, but I'll bet a large, round American dollar that they can't do it. The system is too perfect and its entrenchments too formidable. Besides, what's the use? The sheep would seek a new collar with a brand new stamp upon it long before they had gotten well rid of the old. Selfish human nature on the one side and plastic human nature on the other would merely enable history tc repeat itself. Even if the sheep should wake up, they'd straightway fall asleep again.

And yet, the "looking backward" story said that the dog finally bit off his overgrown, perniciously active tail, so I must not be too pessimistic, although I suspect the poor dog was very sick at his stomach for awhile.

Item: Don't blame that official board of days agone who went so far afield and into the sage brush for an eclectico-homeopathic servant who grew and grew till now he is King of the Amalgamated Association of Medical Tin Gods, Limited. You can't make a whistle out of a pig's tail nor a silk purse out of a sow's ear. Neither shall ye gather figs of thistles nor hatch literary lions from asses' eggs, e'en though they be sugar-coated and incubated in a mare's nest.

Hie thee to the Scriptures, O sheep, and what readest thou? "What thou sowest that shall ye also reap."—Selah. Great is "organization," for thereby flourisheth many tin gods.

The big stick that is falling so hard upon patent and proprietary medicine fakers is a godsend to the profession. I would refer anyone who desires to know my attitude on this question to my "Medicine as a Business Proposition," written many years ago. But, all the same, that big stick in the hands of a petty-minded, pinheaded, rancorous individual, who conceals his venom and cowardice behind the hedge of professional altruism and throws mud from the battlements of an editorial sanctum that belongs not to him, but to the men who employ him, is a dangerous and malevolent thing. And when that same big stick is used to crush presumably honest and worthy men who, like the darky, have "done de bes' dey knowed," the reaction of professional sentiment is likely to injure the glorious cause in whose name the bludgeon has been so malevolently used.

Be it undertood that I am in no wise rushing into print for the purpose of fighting the battles of any particular man. The fact that the troubles of certain men serve to point a moral to adorn a tale or emphasize a protest is something for which I am not responsible. Primarily, I stand by no man who in my best judgment is in the wrong, however much the under dog may appeal to my sympathies. If, however, I should become convinced that a man was wrong in a given controversy I would take up the cudgel for him when I saw that he was being unjustly persecuted. Fair prosecution and the trial of a cause on its merits is something to which no right-minded man will object. Persecution and unfair attacks, however, are repugnant to every man who is built on the square.

Persecution made nihilipathy and all the quackopathies—or, at least, persecution ran a close second to professional bigotry and ignorance.

The skulking Indian in the medical woodpile would better beware; the rebound of his little tomahawk may dethrone a tin god or two, or, peradventure, scotch the head of some hero whom we are glad to honor, though loth to worship.

The fact that many great and good men are arrayed upon the side of a given cause, their names appearing high on the roster of the "leaders," their plump nates reposing placidly and protoplasmically on the seats of the mighty, does not prove the philanthropy, purity, justice or grandeur of the cause.

Moral: The higher the monkey climbs the professional ladder, the more he exposes the fact that his pants need halfsoling. N. B.—And the farther away and more diminutive his self-labeled high-browed cranium sometimes appears to the humble philosopher on the ground floor of the temple of fame.

It will be noted that most of the errors, abuses and outrages with which mankind has been afflicted through all the ages have had the weight of distinguished authority behind them. St. Augustine and Cotton Mather were great and good men and of powerful authority, yet the dicta of the one retarded cosmic truth for hundreds of years and the other burned poor old women at the stake for witchcraft. Personally, I am a bit suspicious of "good and great" men as evidence. History does not paint them prettily. Most of them had blood on their fingers. In medicine, most of the good and great men of aforetime were unconscionable scientific bigots who were drunk with ignorance and intolerant prejudices.

And the shades of the medical bigots peer out at us from the nôt too remote past and babble strange babble of "ethics," the whilst heralding their own greatness from the housetops. And they say of the Homeopath, "Thou shalt not consult with him, for he is an imp of outer darkness." And of the Eclectic they cry, "Unclean! Unclean!"

And these bigots were wise in their generation and were great "leaders." Let their successors beware, for perchance some of their "wisdom" is but an inheritance of bigotry from the medical fathers, and posterity may not hold them in high esteem. Not even the letting down of the bars and the admission of the irregular little pigs into the fertile regular clover patch will save their records if they don't watch out.

It is a far cry from damning the abominable patent medicine business and the fake proprietary nuisance and strangling medical independence. Beware lest ye bridge the chasm.

The nearer you get to some great men the weightier seems the name and the more pusillanimous seems the man. His disinterestedness is largely hypocrisy and self-seeking. His humanity is "I," writ large.

Medicine is today busily engaged in shaking off the dogma of infallibility of our medical forefathers. Let it beware of the infallible authority of the medico-political tin gods—especially let it beware of medico-political Pharisaical "bunk."

Are there really any medico-political tin gods? Well, let us see, my son. A handful of doctors recently assembled in Chicago to endorse Governor Deneen. The inevitable preamble and resolutions follow. The preamble began: "We, the physicians of Illinois," etc., etc. Mind you, I quarrel not with the "endorsement" *per se*. The cause was good enough, and I myself publicly endorsed and voted for Deneen, but—

Ever hear of the immortal three tailors of Tooley street? Well, there you are, my boy; there you are.

The Old Guard bragged that it "died, but never surrendered," and then blindly charged into that fateful ditch at Waterloo. There is a medical old guard which, like its ancient prototype, stands in battle array and makes its boast—and there is a Waterloo looming up on the horizon, and there's a big, big ditch waiting for the serried ranks of the medical infallibles. The handwriting is on the wall.

And so be not dismayed, O sheep. There will one day be things a-doing, e'en though it be but a gab-fest Donnybrook fair with plenty of strenuous protest and no results worth mentioning.

In sympathy with the proprietaries? I should say not! The

"ready-made doctor" and his patron proprietary devil with his "stomachine," "kidneyol," "testine," etc., make me tired. Recollect what I once said about those gonorrhea capsules stamped with the maker's name so that he who runs may read? (*Honi soit qui mal y' pense?*) No? Well, surely you remember what I told the profession about that "little joker anti-pain tablet" with its kindergarten hieroglyphics which the layman has learned by heart. You don't? Well, you may be assured that the people who manufacture it haven't forgotten what I said.

Once upon a time I scored a bull's eye on the character of the ads. in the *Journal of the A. M. A.* in an article entitled: "Is the *Journal of the American Medical Association* a Partisan Organ?"

You see, I was really the pioneer "kicker." Time has answered my question; the *Journal is* a partisan organ; and, worse than all, it is sometimes a bully and a scandal monger. Likewise it is a "mace" ready to the hand of its editor—the master of us all—wherewith to crush anybody whose personality or policy does not suit him. A very unsafe censor of medical literature, this man. Scientific work does not appeal to his understanding, nor does honest literary work appeal to his sympathies, although papers from the sacred inner circle are fetich to him. Fortunately, however, some of his staff selections are first class—indeed, the quality of some of them is beyond criticism.

Beware, O arbiter of the other fellow's ink pot! Remember that ancient retributionary rhyme:

*"He digged a pit, he digged it deep; he digged it for his brother.*
*For his great sin he did fall in the pit he digged for t'other."*

In days aforetime the *Journal* was not so virtuous, was it—no more ethical than was its editor in former days? Oh, no; it then needed the fleshpots. When a courtesan no longer needs the money she sometimes turns reformer, and, oh my, the things she does to her still needy sisters! And her morals is of the chilled steel order. She is virtue incarnate and incoruptible—so long as she needs not to dip her own chaste fingers in the fleshpots. But, trust her not—times may change, and then—

"All flesh is as grass, and necessity may transform icewater into rich, warm, red blood that once more has its price."

See the parallel? Oh, consistency thou art a jewel. Don't forget that celebrated ad. from the columns of the *Journal of the A. M. A.*

"Wanted.—A gentleman past middle age who has been incapacitated by a surgical operation for the performance of his nuptial duties would like to meet a lady similarly situated. Address 1001 Journal Office."

Nothing was said about matrimony, so we infer that a Christian Science debauch was in prospect. Anyhow, Colonel Jim Stuart, chief postoffice inspector, must have been asleep at his post.

It seems that morals is a matter of chronology as well as of geography. Matrimonial agents are social pariahs these days, and assignation ads. are refused by even the most respectable newspapers—and what a "respectable" newspaper will not publish the same is pretty tough and shall not see the light.

Don't forget, please, that the editor of the *Journal of the A. M. A.* was once not only a Homeopath, but advertised in the secular press as a specialist in at least three branches of medicine.

And it is the *Journal of the A. M. A.*—same editor, mind you—that is now posing as chief of the Pharisees. It still, I believe, accepts ads. of preparation that it will not allow mentioned in its reading columns—providing the goods are of American manufacture. You may mention urotropin, however, and thus line the foreigner's pockets. Again, "Oh, consistency," etc.

But we must have money, not for scientific progress—the sections do not see much color of the same—but for "organization" at $8000 per! Or is it $10,000? Walking delegates come high in the A. M. A., don't they? And so do political agents. Perchance, however, the glittering chains of organized slavery are cheap at any price—for those who like that particular brand of jewelry. And the price we pay for a man to go about offering insults to the profession in certain localities is pretty steep. Ask some of the physicians of Cincinnati and Louisville.

The most virulent infection that has ever attacked the body social, the worst enemy progress has ever encountered, the black beast that has spit venom and fire and blood upon poor, helpless humanity through all the ages, has been the dogma of infallibility. Time was when we were wont to associate this horrid spectre of the bloody hand, pillory, roasting bee, axe, gallows, dungeon, exile and ostracism with orthodox theology, fondly hoping that it was dying in the stew of its own corruption. Let us hope that the monstrous all-devouring thing has not sprung to life again under a mask most deceiving and with arguments most wily and plausible. Let us hope that the infallibles of the A. M. A. will not eventually prove to be lineal descendants of a long line of bigots and stranglers of intellectual individuality, and let us further hope that they will not one day impale us on a pin and stick us on the wall that our impotent struggles and ineffectual squawks may make a holiday for pharmaceutical Neros. I confess to some misgivings.

The uncrowned king of the pharmaceutical infallibles was trained as a Homeopath, took a course in Eclecticism, wrote criticisms of regularity and defenses of homeopathy, and, even in the days of the apostatic epoch of his life, had so little therapeutic experience that even his alleged reform and renegadery were a colossal joke. And, *mirabile dictu,* he once advertised himself as an omnibus specialist in the secular press. Let him challenge this statement, if he dares. *I speak "by the card."*

*This also is the man who once wrote, for a journal he has since damned and doubly damned: "Those who run homeopathy down know least about it. The practice of the allopath is to give as much as the patient can stand. * * * I believe in homeopathy."** Can the leopard change his spots?

The infallibles have a high purpose. We admit it. Their ostensible aims and objections are for the most part praiseworthy. Infallibility? We concede it, and now we would fain make demands upon it. When the infallibles have finished with their list of legitimate things, let them give us a list of the official drugs and preparations which can be relied upon for therapeutic action—a list of those drugs whose effects on the human economy are other than negative or disastrous. Seventy-five per cent of the stuff which is endorsed by one or another authority is pharmaceutic and therapeutic rubbish and the stuff that is written about it is simply "bunk" unadorned—a "scientific" feast of the Barmecide. Even the proprietaries could not make a much worse showing. The *raison d'etre* of the proprietary remedy, evil though it often is, is not all greed—the worthlessness of most "legitimate" drugs and preparations is the cornerstone of quackery.

There is much room for honest differences of drug opinion. Wonder what the erstwhile king of therapeutic nihilism, Osler, thinks of the infallible list of official drugs. If his creed is right it would seem that the work of the council was love's labor lost. If he is right the chief danger lies in the legitimate things, rather than in some rather palatable proprietaries.

And by what process of mental "flip flap" does the chairman of

---

*"Medical Ethics," by George H. Simmons, M. D., Medical Brief, April, 1883, p. 168.

the council participate in its profoundly philosophic meditations and erudite decisions? Why, he once wrote ardent "defenses" of homeopathy! (See Medical Brief, April, 1883, p. 168.) Can it be possible that during a few weeks' sojourn—or was it merely a matriculation—at a regular school in the days when those temples of learning "went the Creator one better"—for He only made a man out of mud while the colleges once made physicians out of any old thing—he became a therapeutic scientist so profound that, many years after he had seen his last luckless patient, he was capable of determining the reliablity of "allopathic" drugs and simples? But I forgot; the gentleman is also said to have attended a course in eclecticism, hence he possibly is broadly informed in therapeutics. Then, too, he was once an omnibus specialist in an advertising "institute." Speaking of the prudery of reformed courtesans, why—who and what is this Daniel come to judgment?

I wonder how long it really took the editor of the *Journal of the A. M. A.* to get a "regular" diploma. It could be done in those days in short order, for most of our colleges then belonged to the *monotremata,* their *primæ viæ* being so short and straight that no sooner was a student swallowed than he was excreted, diploma in hand. I believe I'll look this matter up.*

The Bible says that there is more joy in heaven over one sinner who repenteth than over many who have not sinned, but if the official board before mentioned thought of the scriptures at all they forgot that the A. M. A. could not justly claim any heavenly attributes, hence the scriptures had little bearing on filling its editorial vacancies, and the sanctum sanctorum of the *Journal* should not have been made a haven of refuge for a once advertising apostate Eclectico-Homeopathic specialist in diseases of the head, trunk, and extremities—the apotheosis of sin against both medical orthodoxy and homeopathy. The high-salaried plums and honors of American medicine would better fall into the laps of those who have never strayed from the virtuous embrace of good Mistress Science.

Some day I'm going to write a comic opera. The chorus will be composed of nice old medical he-ladies in long pantalettes and lingerie with tatting edges. And they shall all be members of the sacred inner circle and shall caper nimbly to the beating of a big,

---

*I have since found out how long it took him and will later enlighten the profession on that point.—L.

big stick. In the center of the stage shall be a medical editorial bi-salaried Pooh Bah with a much tarnished tin crown.

Do they thusly caper? Come hither, my son, and lend me thine ear:

There was to be a discussion at a certain physicians' club—it seems to be a "club," all right—on the methods of teaching therapeutics. The name of one of America's most distinguished authorities on therapeutics—the author of one of our best standard works—was proposed as a speaker on the programme. Certain members of the board of directors opposed his selection. They "liked him personally," they said, but there would be a lot of "criticism," etc.—"etc." in this instance being a lot of hypocritic balderdash.

And now for the milk in the cocoanut: The gentleman to whom a certain two of the directors were opposed is associated with and friendly to a man whom the editor of the Association journal has gone out of his way to traduce, vilify and to attempt to ruin. Further, the ex-homeopathic editor of the *Journal* was to appear on the proposed program and orate on "the present status of therapeutics!" Alas, poor king of France who with all his men did march up the hill and down again.

Poor, pitiful "hitchers" on to a wobbly chariot; adoring satellites of a star of evil omen; brace of nice old he-ladies of the directory, let me whisper something in your ear: There is more clean sentiment, more high professional ideals, more ethical spirit, more gentility, more breadth, more true culture and more knowledge of therapeutics in one cubic millimeter of the brain matter of that particular man whose name you turned down than in the entire gray matter of both of you. More than this, he is an adornment to the medical profession of which it may well be proud. Of course, some people can not understand such a man—he would be less of a man if they could.

Great Scott! How I do despise a yellow streak. To think that men of supposedly high ideals and intelligence could be so narrow-minded as to allow prejudice that has trickled down from the muddy editorial ink bottle of the *Journal of the A. M. A.* to sway their judgment in a matter of such vital interest to the members of a supposedly independent association of physicians! And this is the twentieth century! Faugh, it makes one seasick!

Really, my therapeutic friend ought to be more congenial with the average medical old he-lady. 'Tis very simple: All he would have to do would be to repudiate the man on whom the *Journal*

has poured the vials of its wrath—repudiate the man who has befriended him and assisted him in the nimble chase for bread and butter, the bread and butter in the getting of which the he-ladies above mentioned would not assist him were he and his family starving.

I trust my good friend will not gather wormwood from the incident I have related. The petty animosity of narrow, picayunish minds toward men of independent thought is the pabulum upon which true greatness fattens. Then, too, other and less brilliant and capable men have gone unscathed through similar experiences.

It makes me laugh when I recall that five old director he-ladies rejected my own application for membership in that same club many years ago. These men were then and are now unknown to fame and, though they should live till the hinges of hades congeal and Gehenna becomes a place for cold storage and skating rinks, yet will the trump of fame not sound for them.

But softly, I forgot: One of those directors of days agone, an ex-preacher of the gospel, who had "reformed," became famous awhile ago through having been robbed of $5000 by a gang of wire-tappers who lured him into a scheme for getting rich quickly by fleecing others. The would-be fleecer was fleeced—and he squealed so loudly that his porcine "vocalics" were heard in high heaven's vault.

"The mills of the gods grind slowly, but they grind exceeding fine." The time came when the original club, through many similar "exclusions," was dying of cerebral paralysis, the etiology of which was dry rot. It was saved from utter extinction by amalgamating under the old name with a club of "live ones." Since then it has done much good and has been the center of considerable intellectual and social activity. Having served as a director of the club since I was "amalgamated" and many times taken an active part in its meetings, it would grieve me to see it become the agent of the tin gods and whacking at the heads of men of brains and capacity who do not happen to be *persona grata* with the oligarchy.

Messieurs the directors, don't you know that the black ball and the mace have never been very effective weapons with which to assail professional gentlemen from ambush? Why, a member of a recent directory of the club in question succeeded many years ago in getting a certain man blackballed five times by the Chicago Medical Society. "The stone which the builders rejected"—that man is now one of America's most eminent surgeons and has served as its president the society which so often repudiated him in the long ago. The gentleman with the blackball habit—and, come to think, he once got me blackballed in a certain national society—is right where he was in the days of his blackballing activity, only a little more so. He has practically stood still since he sprang into the limelight by engineering the notorious, ill smelling abortion exposé in the *Chicago Times* many years ago.

And now, messieurs the particular directors, for whom this gentle criticism is intended, let me give you a bit of friendly advice: Don't fool away your time hitting at the heads of men who think their own thoughts. Resolve yourselves into a scandal club—a sewing circle—make panties for the Fiji Islanders and embroidery for the Esquimaux, crochet warm mufflers for the Ashantees and talk about the neighbors. Thus will you avoid doing harm to progress and fall into the place in the social scheme for which nature originally designed you.

Heavens! What have I done? The directors afterward changed their minds—or their policy, which less begs the question—and my therapeutic friend was put on the program. Really, I have hopes—not of the reform of the tin gods, but that the fears of their servants and servile satellites may help some—especially if the real men of the directory take a hand.

The world will roll on in the lathe of time despite the creaking of narrow minds. Meanwhile, let us hope and let us dream of the medical sweet by and by, when great medical associations will be democratic, when the editors of official organs will have clean hands and will be fair and will not prostitute their high office to the attainment of private ends and the gratification of personal spite in endeavoring to bring discredit or even financial ruin upon a fellow member of the Association by going beyond their province, using valuable space belonging to the Association and partisanly discussing the private affairs of a man who would have received no attention had he not been the victim of the wrath of the tin gods.

---

"BACKBONE" is the title of a little book by Dr. S. DeWitt Clough, of Chicago, a compilation of brief, breezy and uplifting articles. Its aim (and effect) may be summed up in one word: "Optimism." Dr. Clough calls it "Hints for the prevention of jelly-spine curvature and mental squint: A straightup antidote for the blues and a straight ahead sure cure for grouch." Price, 50 cents.

## Figures and Tables

**Figure f1:**